# Encoding of 3D physical dimensions by face-selective cortical neurons

**DOI:** 10.1073/pnas.2214996120

**Published:** 2023-02-21

**Authors:** Amit P. Khandhadia, Aidan P. Murphy, Kenji W. Koyano, Elena M. Esch, David A. Leopold

**Affiliations:** ^a^Section on Cognitive Neurophysiology and Imaging, Laboratory of Neuropsychology, National Institute of Mental Health, NIH, Bethesda, MD 20892; ^b^Neurophysiology Imaging Facility, National Institute of Mental Health, National Institute of Neurological Disorders and Stroke, National Eye Institute, NIH, Bethesda, MD 20892; ^c^The University of Colorado Anschutz Medical Campus, Medical Scientist Training Program, Aurora, CO 80045

**Keywords:** visual objects, inferior temporal cortex, macaque, naturalistic behavior, face processing

## Abstract

Neurons throughout the primate inferior temporal (IT) cortex respond selectively to visual images of faces and other complex objects. The response magnitude of neurons to a given image often depends on the size at which the image is presented, usually on a flat display at a fixed distance. While such size sensitivity might simply reflect the angular subtense of retinal image stimulation in degrees, one unexplored possibility is that it tracks the real-world geometry of physical objects, such as their size and distance to the observer in centimeters. This distinction bears fundamentally on the nature of object representation in IT and on the scope of visual operations supported by the ventral visual pathway. To address this question, we assessed the response dependency of neurons in the macaque anterior fundus (AF) face patch to the angular versus physical size of faces. We employed a macaque avatar to stereoscopically render three-dimensional (3D) photorealistic faces at multiple sizes and distances, including a subset of size/distance combinations designed to cast the same size retinal image projection. We found that most AF neurons were modulated principally by the 3D physical size of the face rather than its two-dimensional (2D) angular size on the retina. Further, most neurons responded strongest to extremely large and small faces, rather than to those of normal size. Together, these findings reveal a graded encoding of physical size among face patch neurons, providing evidence that category-selective regions of the primate ventral visual pathway participate in a geometric analysis of real-world objects.

## Significance

Object-selective neurons in the inferior temporal (IT) cortex are modulated by changes to image scale, although the essence of such modulation is unknown. Here, we asked whether the two-dimensional (2D) or three-dimensional (3D) geometry of an object is the critical factor. We used a 3D macaque avatar rendered stereoscopically at different size and distance combinations to independently assess the contributions of retinal and physical size parameters. We discovered that neurons in a face-selective region of IT are strongly modulated by 3D physical size. This finding demonstrates that IT neurons encode metric and spatial parameters, in addition to semantic and featural information.

We experience the world in three-dimensional (3D) space, perceiving and interacting with objects and individuals in a scene. For humans and other primates, much of this experience is served by vision, with broad stretches of the cerebral cortex ostensibly devoted to making visual sense of the world. For example, individual neurons throughout the inferior temporal (IT) cortex of the macaque respond selectively to meaningful objects, with neurons of similar response properties often aggregated in functional clusters (1–3). One striking finding about the object selectivity of IT neurons is its tolerance to natural image transformations, such as scaling and translation (4–12). Namely, if stimuli are ranked based on the responses they elicit from a given neuron, this ranking often remains unchanged when stimuli are translated on the screen or scaled up or down several-fold in size. Scale tolerance in object selectivity is thought to reflect the capacity of the brain to compute a conceptual or abstracted representation of the retinal image separate from its metric details. While the mechanism underlying this apparently intrinsic feature of ventral stream visual processing is poorly understood, it is thought to be critical for image-based object recognition (4, 13–17).

At the same time, scaling an image up or down can greatly change the responses of IT neurons to stimuli, even as the rank-order selectivity to stimuli is preserved (18–20). This size-dependent rate modulation is poorly understood and seldom considered explicitly. One relatively unexplored possibility is that some IT neurons encode the physical dimensions of objects, in addition to their shape, and their featural, and semantic properties. The explicit coding of parameters such as absolute object size and distance from the observer might facilitate visual operations concerned with the perception of scene geometry and interaction with the local environment. Additionally, the brain may benefit by retaining internal metric information about the typical sizes of objects (21, 22), as this information could be applied to subsequent perceptual judgments about objects and individuals in the context of natural visual behaviors (23).

The visual encoding of 3D space is usually associated with parietal cortex in the dorsal visual pathway, where coordinate transformations are thought to convert retinal signals to 3D information about objects and the environment that can be used to guide effector actions (24). However, a few studies have demonstrated that neurons in the ventral pathway also exhibit signals related to 3D spatial perception. For example, in area V4 neural responses to a given retinal image are modulated based on the physical distance at which that image is presented (25) as well as volumetric 3D shape parameters (26). At later ventral pathway processing stages, the superior temporal sulcus (STS) is marked by selectivity to 3D object shape, potentially reflecting their interplay with intraparietal areas concerned with 3D visual geometry (27–32). While these findings demonstrate that 3D information influences responses across the ventral visual pathway, little is known about whether these areas explicitly encode the physical dimensions of objects, such as their size or distance from the observer.

Here we explicitly investigate how a population of category-selective neurons in macaque IT encode the physical dimensions of objects. We recorded from the anterior fundus (AF) face patch (33), a well-studied face-selective region of the STS where neurons are known to be both selective for faces and sensitive to their spatial scale (34). We asked whether such scale sensitivity primarily reflects the 2D image of a face on the retina or the 3D physical geometry of the face and head in the real world. In most visual electrophysiology experiments, the retinal and physical geometry of an image are yoked: scaling an image on a display alters both its physical size and its retinal subtense. Moreover, absent other explicit depth cues, an image has ambiguous depth and thus cannot be uniquely mapped to the 3D world. In the present study, we used a recently developed macaque avatar model (35) to stereoscopically render photorealistic 3D faces of unambiguous physical size and distance. We found that the size sensitivity of most AF neurons was dictated primarily by the physical dimensions of a face rather than by its angular subtense on the retina. We further discovered that neural responses were strongest to extreme-sized faces rather than normal sized faces, opposing intuition but consistent with ideas of predictive coding. We discuss how object-selective IT neurons might contribute to important and conserved elements of natural visual behavior through their encoding of real-world geometric parameters.

## Results

**Fig. 1.**
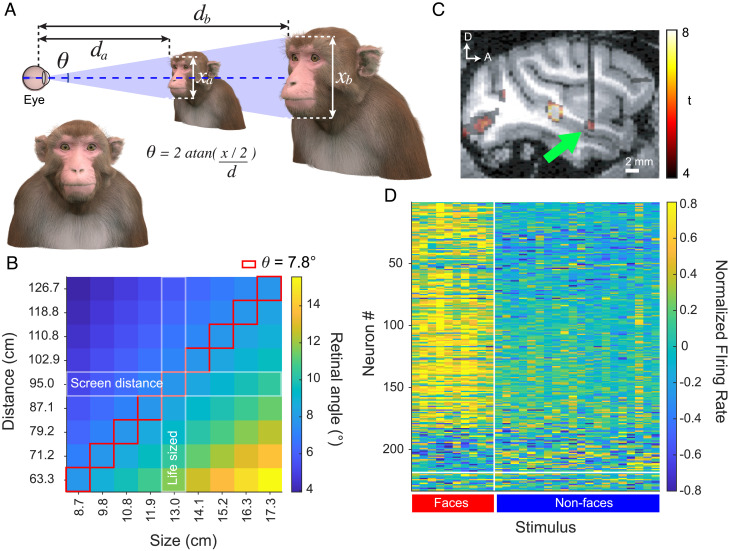
Macaque 3D avatar stimulus scaling and retinal angle matching. (*A*) An example of the macaque avatar rendered in 2D and a schematic representing retinal subtense matching in 3D space where d represents the virtual distance between the subject’s retina and the stimulus, while x represents the size of the presented face and θ is the retinal angle as determined by the formula, which is equal between the two stimuli displayed here. (*B*) Heatmap displaying the retinal angle of all size and distance combinations with the red outline indicating the stimuli with equal retinal angle and the white outlines indicate the screen distance and the average size. (*C*) An fMRI overlay of the localization and targeting of the AF face patch in one subject, with an arrow indicated the position of the microwire bundle. (*D*) Heatmap of the responses of all recorded neurons where each row represents a neuron and each column a stimulus. The heatmap is separated into face-selective and non-face-selective neurons by the horizontal white bar and face and non-face stimuli by the vertical bar.

To investigate whether the size modulation of AF neural responses is determined more directly by the angular or physical size of a face, we stereoscopically presented nine different sized renderings of an avatar face at nine different distances (Fig. 1 *A* and *B*). We placed particular focus on the responses to a subset of size/distance combinations that yielded images with equal retinal angle (Fig. 1*B*, red outline).

**Fig. 2.**
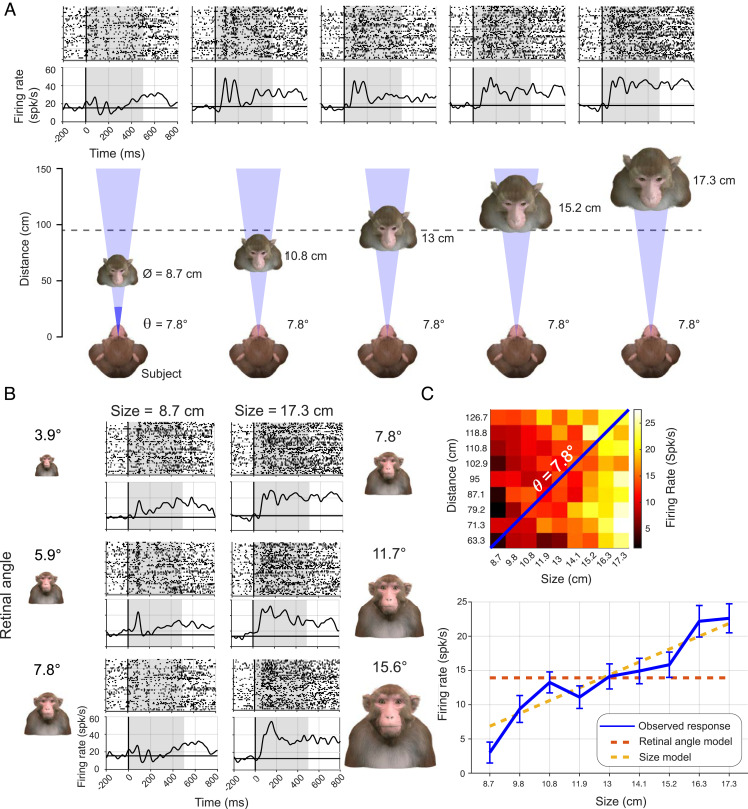
Example response of a physical size tuned neuron. (*A*) Responses of an example neuron to five stimuli with the same retinal angle but different physical size distance combinations displayed in the schematics below. Responses are shown in in spike rasters (*Top* *Row*) and spike density functions (*Bottom* *Row*). (*B*) Responses of the same example neuron to stimuli with different retinal angle while each column represents a constant physical size of either the smallest (8.7 cm) or largest presented (17.3 cm), demonstrating that physical size played a stronger role than retinal angle in the size tuning of this neuron. (*C*) Average response of this example neuron across all the different physical sizes of equal retinal angle stimuli (blue) compared to the physical size model (yellow) and retinal angle model (orange). The heatmap above shows the response profile for all 81 size distance combinations with equal retinal stimuli indicated in with the blue diagonal.

**Fig. 3.**
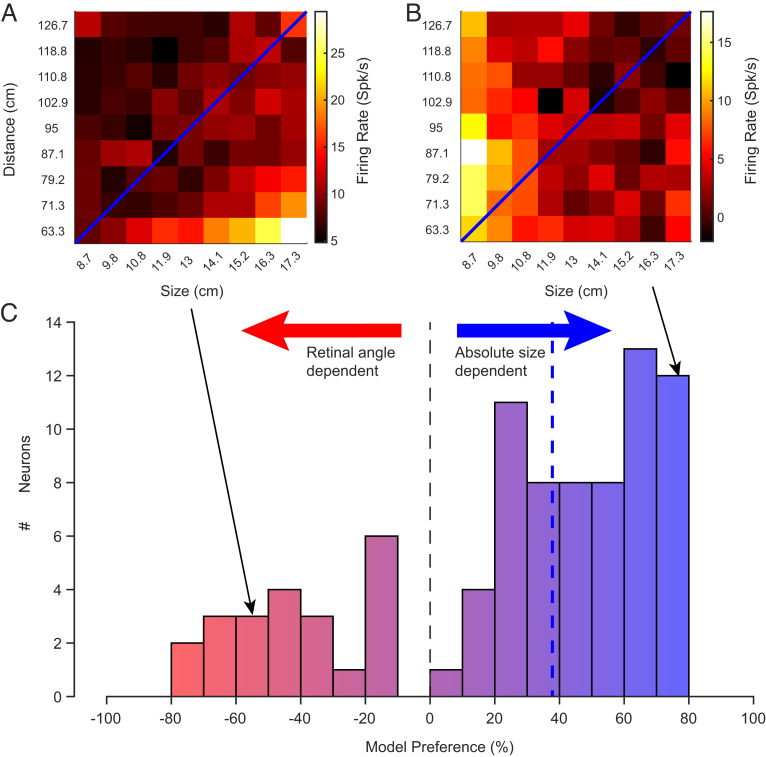
Model preference for physical size or retinal angle across the neural population. (*A* and *B*) Heatmaps of the response of two example neurons that prefer retinal angle (*A*, model preference = −56.1%) or physical size (*B*, model preference = +73.4%). The blue diagonal indicates all the stimuli with equal retinal angle (*C*) Histogram mapping the model preference of the whole neural population (see main text and *Methods*), with arrows indicating the dependence on absolute size or retinal angle and the dashed line displaying the median of the population. Across the population, most neurons responded were more sensitive to absolute size over retinal angle.

**Fig. 4.**
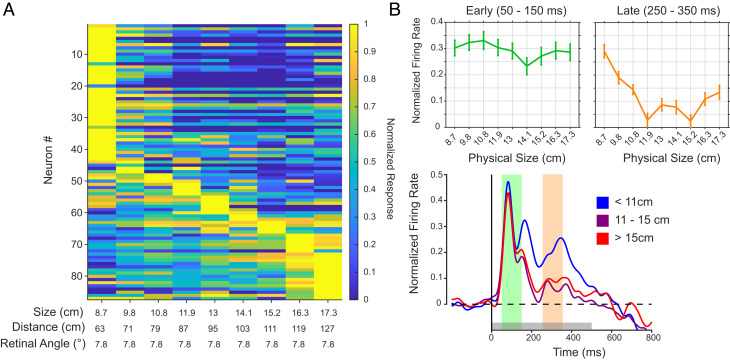
Size preference of neural responses. (*A*) Heatmap of the normalized response to stimuli of equal retinal angle (7.8°) where all responses are normalized to largest mean response across stimulus presentation. (*B*) The average time course with the smallest sizes in blue, the middle sizes in purple, and the largest in red with average tuning curves for the designated time window indicated by the colored shading. All firing rates were normalized to the peak firing rate for each neuron. Beyond showing sensitivity to physical size, the results revealed that most neurons preferred the largest or smallest physical sizes of the avatar face particular in the later phase of viewing.

**Fig. 5.**
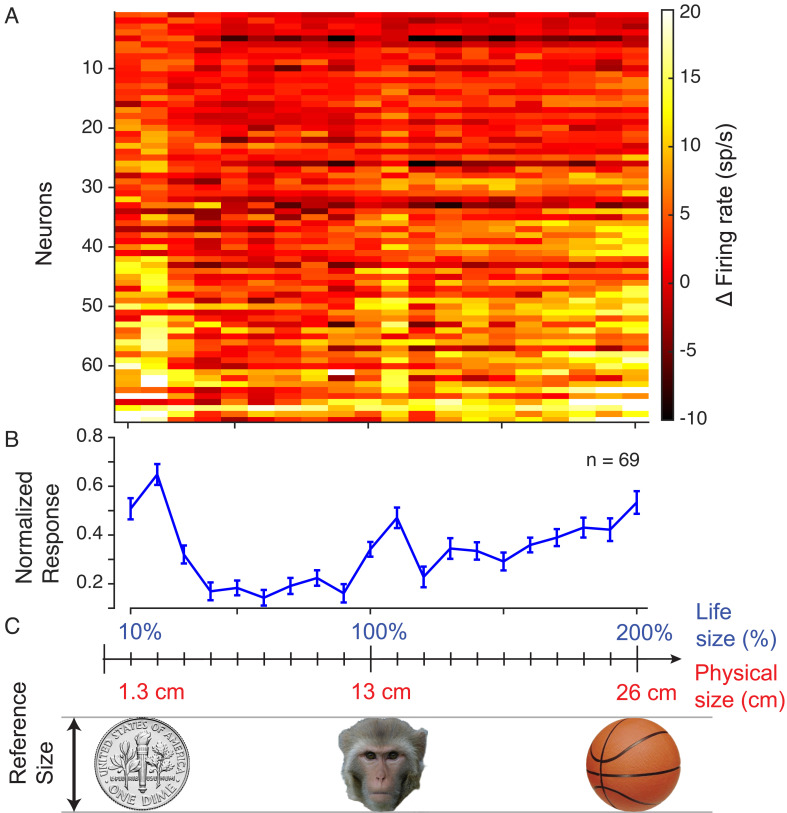
Neural response to extreme macaque face sizes. (*A*) Heatmap of the raw firing rates of the neural population across all 20 sizes shown. (*B*) Mean normalized response of the neural population across all 20 sizes showing the strongest responses for the extreme sizes. (*C*) Size of 3D avatar face stimuli relative to real-world objects.

The activity of a total of 354 neurons was recorded from the AF face patch of two adult rhesus macaques (129 from male Monkey SR and 225 from female Monkey Sp, Fig. 1*C*). Of these 354 neurons, 114 did not meet the visual response criteria of greater than 3 spks/s above baseline to at least one of the rendered avatar images and were thus excluded from further analysis. For each neuron, we also presented a different image set from which we computed the face-selectivity index (FSI, see *Methods*). We classified cells as face-selective if they had an absolute FSI greater than 0.333, corresponding a doubling of response to faces compared to non-face stimuli. Of the 240 neurons meeting response criterion, 90.8% (218/240) were face-selective (Fig. 1*D*), 87 were tested in the first experiment of physical size tuning (Figs. 2–4), 69 were tested in the second experiment of preferred size (Fig. 5), and 84 were tested in the additional experiment using non-face objects (*SI Appendix*, Figs. S3 and S4).

### Neurons Sensitive to Physical Size of the Face.

In the first experiment, we tested the responses of 87 neurons, 89.7% (78/87) of which were face-selective, to a stimulus set of 81 images at nine sizes and nine different distances resulting in nine stimuli with equal retinal angle but distinct size and distance. Neural responses were markedly affected by the physical geometry of a face stimulus, even when its retinal angle remained unchanged. This effect is illustrated by the responses of an example neuron to a stimulus of fixed identity in Fig. 2. When the retinal angle was fixed at 7.8° but the physical size and distance of the stimulus were stereoscopically varied, the visual response magnitude was strongly determined by the physical size of the face and head (Fig. 2*A*). On the other hand, holding the physical size constant and varying the distance/retinal angle (i.e., left or right column in Fig. 2*B*) caused only a modest size modulation in the same neuron, even when the retinal angle varied by a factor of two. Fig. 2*C* shows responses to the subset of stimuli sharing a 7.8° retinal angle, comparing the observed responses to the predictions of the retinal angle model (which would predict no change) and the physical size model (which would predict monotonic change). The responses of this example neuron clearly match the expectations of the physical size model.

Across the population, most neurons (81/87) were significantly modulated by physical size, with a partially overlapping subset (60/87) significantly modulated by physical distance (two-way ANOVA, *P* < 0.01). Importantly, 63.3% (56/87) of neurons were sensitive to the specific physical size/distance combinations that rendered the same retinal angle (one-way ANOVA, *P* < 0.01). These findings indicate that the responses of AF neurons are strongly shaped by the real-world geometrical parameters of the face, even when the corresponding visual images have the same retinal angle.

To quantify and compare the relative contribution of physical size versus retinal angle across the AF neural population, we performed a linear regression analysis to determine the factor having the strongest influence on the responses of each neuron. For the response matrix of all size/distance combinations, we compared linear regression models using retinal angle or physical size parameters alone, to a multiple regression model including both factors (for details, see *Methods*). This method provided a means to assess the preference for the retinal angle versus physical size model in capturing the variance of the data under conditions in which the two parameters have considerable covariation (36). For this analysis, the model preference could range from a value of −100%, indicating complete dominance retinal angle parameter on neural responses, to +100% indicating complete dominance of the physical size parameter. When this analysis was applied to the example neuron in Fig. 2, the model preference was +62.1%.

Across the population, the majority of neurons registered positive values, indicating that their responses were more strongly determined by physical size than retinal angle, mirroring the ANOVA results above (Fig. 3). Specifically, 74.7% (65/87) of neurons were driven more strongly by the physical size of the faces, whereas only 25.3% (22/87) of neurons were driven more strongly by angular size of the faces. The median index of 37.8% (*P* < 0.0001, Student’s *t* test) for the neural population indicated that, on average, the physical size accounted for more than double the variance of the neural response as compared to the retinal angle model. This result was similar when analysis was restricted to face-selective neurons (*SI Appendix*, Fig. S1). A model preference comparison of physical size and distance also demonstrated that 74.7% (65/87) of neurons were driven more strongly by physical size than distance. These results indicate that the physical size of a face is a stronger determinant for the responses in the AF face patch than the retinal subtense of the corresponding image, indicating these neural responses are rooted in the 3D physical geometry of faces.

### Neurons Respond Most to Extreme Physical Sizes.

We next examined the size tuning profiles of the same population of neurons in greater detail to determine if there was a bias to prefer particular sizes across the population. For this, we restricted our analysis to the size/distance combinations having the same retinal size. This analysis unexpectedly revealed that neurons were rarely tuned to the real-world size of the macaque face and head, but instead responded most strongly to the smallest or largest sizes tested (Fig. 4*A*). Of the neurons, 58/87 (66.7%) displayed their greatest response to the largest or the smallest sizes, while only 5 (5.8%) neurons responded most strongly to the average or real-world size of a face of 13 cm. These results indicate that physical size tuning is a prominent feature if AF neurons, but this tuning is to the relatively large and small sizes of faces rather than to its most common size.

The tuning to extreme sizes emerged gradually following the presentation of the stimulus and was most obvious during the late response period. This trend is visible in the mean neural responses time courses (colored traces in Fig. 4*B*), which demonstrate that the stronger response to the largest and smallest physical face sizes first emerged 150 ms following stimulus onset. The physical size tuning curves during early and late epochs further demonstrate that the tuning to extreme sizes was fully expressed after 250 ms (Fig. 4 *B*, *Upper*).

To investigate whether this preference for extremes extended beyond the sizes we tested initially, we conducted an additional experiment in both monkeys in which we substantially increased the range of physical head sizes. For this experiment, the stimuli were rendered at a 90 cm viewing distance, corresponding to the physical position of the display. The subjects viewed avatar face at 20 different sizes ranging from 1.3 cm (one-tenth the size of an average macaque face) to 26 cm (twice the size of an average macaque face) at intervals of 1.3 cm. We recorded from an additional set of 69 neurons to test responses to these stimuli.

Despite the marked size deviation from a normal macaque face and head, neurons across the population continued to respond most vigorously to the extreme-sized stimuli (Fig. 5 *A*–*C*). Most individual neurons responded strongest to either the large or small extreme sizes, with some neurons responded to both extremes and less to intermediate sizes. Across the population, 73.9% (51/69) of neurons exhibited their strongest responses to either very small (<=2.6 cm) or very large (>=24.7 cm) face stimuli or both. Neurons with higher firing rate were more likely to be tuned to both extremes as eight of 10 of the highest firing neurons expressed this large and small extreme tuning. These features were also evident in the average population response (Fig. 5*B*). In addition to the preference for extreme sizes, a subpopulation of cells also showed an elevated responses to the 14.3 cm size, possibly reflecting the real-world size of the macaque face. 27.5% (19/69) of neurons showed their maximum response at this size when the largest two and smallest two extreme sizes were excluded. Similar to the tuning within the previous stimulus set, time courses indicated that the tuning to both extremes emerged after the initial presentation (*SI Appendix*, Fig. S2). With the broader size range, the tuning to both extremes emerged approximately 250 ms after the beginning of stimulus presentation, matching the previous stimulus set.

Finally, we asked whether the observed tuning to extreme face sizes would generalize to other rendered 3D objects. To address this question, we presented 12 different objects at nine physical sizes ranging from 1.3 cm to 26 cm (*Methods* and *SI Appendix*, Fig. S3). In this experiment, the virtual distance of the 3D objects matched the screen distance, which was 90 cm. The objects included animals, familiar and unfamiliar fruit, and man-made objects. While the objects were diverse in their real-world sizes (e.g., a fork and a goat), we presented them all at the same range of physical sizes. We recorded responses to these stimuli from an additional set of 84 neurons, 77 of which were face-selective. For each neuron, we analyzed the size tuning for the monkey face, as well the object other than the monkey face (“best-other”) that elicited the strongest neural response at any size. We found that, for some AF neurons, the size tuning matched for face and best-other objects, whereas for others the size tuning differed markedly (*SI Appendix*, Fig. S4 *A* and *B*). For most neurons, the extreme-sized best-other object elicited the strongest responses (53.6%, 45/84; *SI Appendix*, Fig. S4*C*). Interestingly, however, the neural subpopulation showing preference for small macaque faces and that showing preference for large macaque faces were both strongly biased for large renditions of the best-other objects (*SI Appendix*, Fig. S4*D*). Clearly, further investigation is needed to understand the complex relationship between a neuron’s categorical selectivity, responses to objects outside the category, encoding of 3D physical size, and internal representation of an object’s typical real-world size.

## Discussion

The present results demonstrate that object-selective neurons in the inferior temporal cortex are sensitive not only to the identity of objects, their shape, and their constituent features, but also to their physical dimensions. Specifically, the responses of most AF face patch neurons were strongly influenced by the 3D size of a stereoscopically presented face and much less so by the subtense of its retinal projection per se. Unexpectedly, AF neurons responded to life-sized face stimuli with only moderate responses, reserving their strongest responses to extreme sizes, a feature that emerged in the later phase of stimulus viewing.

### The Use of Metric Object Information in Natural Vision.

The brain’s registration of the physical size and other 3D features of objects is fundamental to its ability to interact effectively with the environment (25, 37–40). Nonetheless, relatively few studies have attempted to examine how real-world geometrical parameters might be linked to the basic operation of the ventral visual pathway. Traditionally, vision researchers have considered object size in terms of retina angle, a combination of the physical size and distance of a stimulus, when reporting the effects of size on the tuning of IT neurons (9, 13, 18). Angular subtense is undeniably an important parameter for neurons in early visual areas, where physiological response properties are defined largely by the geometry of the retina. However, this angular measure of size seems unlikely to be the natural domain for the brain’s coding of objects. In fact, several size illusions, some of which have been reported in the macaque (41, 42), provide evidence that the brain does not rely on retinal angle for its internal visual model of object size, even in conditions without stereoscopic cues.

The explicit coding of object and scene geometry in the ventral stream may be important for problems of natural vision that are not typically studied in the laboratory. For example, at the perceptual level, the physical dimensions of objects occupying a scene can help the brain to establish its 3D spatial layout and the corresponding behavioral affordances. For primates, metric object representations are likely to be critical for reaching and grasping movements that are at the heart of their behavioral repertoire, including arboreal locomotion, foraging, and physical interaction with other individuals (43–45). As we learn more details about metric object encoding in the ventral visual stream, it will be important to design experiments that investigate how such signals are utilized by circuits throughout the brain specialized for manual behaviors, social judgements, and scene perception.

### Face Cells Coding Geometric Information.

Few, if any recent studies, have considered the influence of real-world geometric parameters face-selective neurons (19, 20, 46). However, one study conducted more than 35 y ago (19) did find that small subset of neurons (a total of four cells) encoded the absolute size of face images. Namely, when the size and distance of a face image were independently varied, this small subgroup of IT neurons exhibited absolute size constancy, whereas many other neurons did not. This early and largely forgotten observation is likely the same phenomenon observed in the present study. Now equipped with a 3D scalable avatar stimulus, we showed that this sensitivity for physical geometry is an abundant property of neurons in a face-selective region in the STS fundus.

Our findings raise the possibility that the metric sizes of faces, bodies, other objects may figure more prominently into the visual system’s analysis of a scene than previously believed. For example, since heads and bodies are commonly encountered objects that have stereotyped sizes, they might serve as fiducials to interpret the geometrical layout of a complex scene, particularly one populated by multiple individuals at different distances from the observer. The fundus of the STS, where the AF face patch resides, may be particularly enriched with metric representations of objects and scenes. Beyond containing multiple face and body patches, fundal STS regions are thought to constitute a pathway specialized to support dynamic social interactions (47), which, in the real world, are usually constrained strongly by spatial information.

A striking result from our study is that neurons responded most strongly to extreme physical sizes, double the size or a tenth the size of a typical face. The AF population was marked by an intermixing of neurons favoring small and large sizes, as well as many neurons responding to both extremes. This consistent finding might suggest that responses may be governed by deviations from an expectation, a coding principle commonly known as prediction error (48). This framework may explain the discovery of more cells tuned to both extremes in the second experiment as compared to the first, as more unexpected sizes were presented. In that sense, the current findings parallel studies of face identity, where the most extreme face identities give rise to the strongest neural responses, including among AF neurons (49–52). For both size and identity, tuning to the extremes emerges late in the visual responses, possibly reflecting the dynamic suppression of the expectation, or alternatively reflecting a delayed attention to extremes, implemented by feedback from other brain areas (53). The origin of this late modulation, and its basic nature vis-à-vis internal prediction, attention, familiarity, geometrical context, or other cognitive operations, clearly warrants further investigation.

### Spatial Representation in the Ventral Visual Pathway.

The current findings add to several recent studies that appear to contradict the conventional view that the ventral visual pathway is principally dedicated to the recognition of objects through the analysis of shape. For example, our findings on physical size tuning complement electrophysiological studies that have revealed an unexpectedly broad range of geometrical scene statistics encoded explicitly in the ventral visual cortex. Specifically, Connor  et al. (46, 54) found that some IT neurons were tuned to massive 3D shapes, whereas others were tuned to planar or immersive scene geometries. Strikingly, they also found that IT neurons showed tuning for objects at particular orientations, but that these orientations were not defined in retinal coordinates but in a gravity-aligned reference frame relative to the 3D layout of the background scene. These findings, like those of the present study, suggest that object representation in IT is closely connected with the broader geometry of scene elements and determined by the natural statistics of the world. At the other end of the spectrum, recent work has indicated that object-selective responses in the ventral visual pathway, including face-selective responses in area AF (55), are dictated by highly specific and local features, rather than by global shape or holistic configurations (56).

An emerging view holds that object representation in the ventral visual pathway is significantly influenced by its communication with parietal areas (reviewed in ref. 56). Previous electrophysiological recordings in the STS have suggested a direct interaction of visual object areas with parietal regions supporting visually guided manual behavior (27, 28). Such interaction may be mediated directly or indirectly through connections between the STS and visuomotor parietal cortex (57–59). Parieto-temporal interaction is further supported by functional magnetic resonance imaging (fMRI) experiments demonstrating modulation of visual activity by stereoscopic 3D structure, including STS face patches (60). These regions of the STS are not strongly modulated by vergence eye movements, which covary with stereoscopic depth, but instead appear genuinely sensitive to 3D structure (61). Whether input from parietal areas or elsewhere contributes to the metric size tuning reported in the present finding, the interplay between dorsal and ventral pathways is almost certainly a critical feature of brain operation during interaction with objects and individuals during natural behavior.

Some evidence suggests that metric information simultaneously influences visual responses at multiple stages of the visual hierarchy. For example, neurons in early stages in areas V1 and V4 alter their responses to the same retinal stimulus when the physical distance to the display is varied (25). Likewise, size illusions that cause a given stimulus to appear at different distances can alter fMRI responses in V1 (25, 37). The origin of such modulation is unknown but may reflect feedback from higher-order regions of the cortex, including parietal areas or areas specialized for object vision (38–40). Likewise, explicit metric information computed in, or imposed upon, early visual areas may influence downstream regions in the STS and elsewhere, where neurons are known to encode the 3D structure of objects (30, 62) based on both monocular and stereoscopic depth cues (63, 64). Thus, metric information may be embedded at multiple cortical levels into the fabric of the brain’s internal representation of objects and scene composition.

Finally, previous fMRI studies in humans have indicated that real-world size, namely the typical physical size of a known object, is stored inherently in the brain and may serve as an organizing principle of the visual cortex (65). Though coding for physical size reported in the present study is distinct from that for real-world size reported previously, the two variables are linked in that both depart from the retinal coordinate framework and take into account the physical size of objects in the real world. Understanding the connection between these variables, namely how neurons reconcile object selectivity, physical object size, and real-world object size, is an important but challenging problem. At minimum, it will require systematic investigation of neural responses to a collection of inherently large and small objects in different categories, each presented at a range of 3D sizes and distances. Nonetheless, this topic is of great importance to understand the nature of high-level visual specializations among cortical areas in the STS or elsewhere and may provide another lens to understand the functional specialization of these regions. These cortical areas are believed to support social interactions and other forms of natural visual behavior, many of which evolved to operate based on highly defined spatial parameters.

## Materials and Methods

We conducted electrophysiology recording in two macaque monkeys. Both macaques underwent a face patch localizer to target and implant AF face patch with a chronic microwire electrode bundle. These bundles were advanced into the AF face patch following implantation until stable neural recordings were achieved. All procedures were approved by the Animal Care and Use Committee of the National Institute of Mental Health and were conducted in accordance with the National Academy of Sciences Guide for the Care of Laboratory Animals and the NIH Animal Research Advisory Committee Guidelines. The NIH Animal Care and Use Program is accredited by the Association for Assessment and Accreditation of Laboratory Animal Care (AAALAC), International.

For the main experiments, we recorded from AF neurons as subjects viewed three different sets of 3D stimuli across multiple sessions (each stimulus set was presented separately from the others). All stimuli were rendered in 3D using Blender 2.79 and presented on a 3D TV monitor. All stimulus sets included renderings of a 3D macaque avatar rendered at different sizes enabling full control in 3D space of a face stimulus. The first set of stimuli included nine different physical sizes ranging from 8.7 cm to 17.3 cm and nine different virtual distances ranging from 63.3 cm to 126.7 cm with a subset of nine stimuli rendered to occupy equal retinal angle but from different size distance combinations. The second stimulus again featured the avatar stimulus but rendered only at one virtual distance but 20 sizes ranging from 1.3 cm to 26 cm to explore the limits of neural size tuning. Finally, the third stimulus set included nine sizes of the macaque avatar ranging from 1.3 cm to 26 cm again but also included size-matched object stimuli including familiar objects such as apples and bananas to unfamiliar such as a fork or a house (*SI Appendix*, Fig S3). All stimuli were presented for 500 ms with at least 500 ms intervals between stimuli.

All data were analyzed and evaluated offline using custom software written in MATLAB. All spike sorting was conducted offline using the wave_clus spike sorting algorithm (66) utilizing resources from the NIH High Performance Computing core. Following sorting, we calculated the mean spike rate of neurons during stimulus presentation and subtracted the baseline spike rate from the response to evaluate the differences between stimuli. For normalized time courses and tuning curves, we calculated the normalized firing rate by normalizing the response across neurons by dividing the response during stimulus presentation by the peak response across all stimuli. Normalized responses were also calculated by dividing the strongest mean baseline-subtracted response across the 50 to 500 ms time window from all mean responses to stimuli. To evaluate the significance of responses, we utilized a combination of two-way ANOVA and one-way ANOVA to determine significant differences of spike rate to different stimuli.

We also evaluated the relative contribution of physical size and retinal angle to the response of neurons to the first stimulus set using the measure of model preference. Briefly, model preference compared a linear regression model across all 81 stimuli for physical size or retinal angle separately to a model combining the two. First, we calculated the model fit for each factor independently comparing the reduction in deviance of a single factor model to the reduction in deviance of combined model. This calculation generated a model fit for physical size and retinal angle independently. We then subtracted the model fit of retinal angle from model fit of physical size to obtain model preference such that positive preference indicated that physical size explained more of the neural response and a negative preference indicated that retinal angle explained more of the variance.

More details are available in *SI Appendix, Materials and Methods*.

## Supplementary Material

Appendix 01 (PDF)

## Data Availability

Data and code to make figures data have been deposited in Figshare (https://doi.org/10.6084/m9.figshare.​20522892) (67).
