# Supplementary material for: Encoding of 3D physical dimensions by face-selective cortical neurons
Source: Proc Natl Acad Sci U S A. 2023 Feb 21;120(9):e2214996120. doi: 10.1073/pnas.2214996120 (PMC9992780; doi:10.1073/pnas.2214996120)
Supplement: Supplementary file 1 — Appendix 01 (PDF) [file pnas.2214996120.sapp.pdf]

Supplemental Materials and Methods

Subjects

Two rhesus macaque monkeys, designated SP (Monkey 1, Female, 18yrs, 9 Kg) and SR (Monkey 2, Male, 6yrs, 10 kg), were implanted with a chronic microwire electrode bundle in AF face patch held by a custom MRI compatible chamber and microdrive. Electrodes were advance towards the target recording depth post-surgically to achieve stable recordings. Monkey SP was implanted in the right hemisphere while Monkey SR was implanted in the left hemisphere. All procedures were approved by the Animal Care and Use Committee of the National Institute of Mental Health and were conducted in accordance with the National Academy of Sciences Guide for the Care of Laboratory Animals and the NIH Animal Research Advisory Committee (ARAC) Guidelines. The NIH Animal Care and Use Program is accredited by AAALAC, International.

fMRI

All functional and anatomical magnetic resonance imaging (MRI) was conducted in the Neurophysiology Imaging Facility Core (NEI, NIMH, NINDS) in a 4.7T Bruker Biospin scanner. Subjects underwent a face-patch localizer to enable targeting of face patches. For all functional scans, subjects received an injection of monocrystalline iron-oxide nanoparticles (MION) to augment hemodynamic response. Monkey SP underwent a standard block design consisting of 24 s blocks composed of images of static macaque faces contrasted with blocks of images of non-face objects [1]. Monkey SR viewed a dynamic localizer, which contrasted blocks of clips of macaques making facial expressions with clips of moving scenes or moving objects [2]. The subject was rewarded every 2s for consistent fixation. All fMRI data were analyzed using AFNI and custom software created in MATLAB we have used in previous studies.

Stimuli

The face stimuli consisted of a 3D realistic macaque avatar presented at different sizes and stereoscopically defined distances (see Murphy and Leopold 2019). Briefly, the avatar was developed using computed tomography (CT) volumes of rhesus macaque monkeys averaged into a single avatar, which received computer generated (CG) texture and realistic fur in the 3D

animation software and were rendered in Blender 2.79 (https://www.blender.org/). For the present study, we used custom Python scripts in Blender to systematically manipulate scale of the avatar with the virtual environment and its distance of the from the virtual camera This enabled us to present the stimuli at multiple physical locations and multiple 3D sizes, in many cases holding the retinal angular subtense constant (**Fig 1A**).

We also presented a series of objects and animals to examine if tuning to sizes was exclusive to faces. We obtained meshes from online repositories that could be manipulated in the Blender software and preformed similar size manipulations to match sizes presented with the face stimuli.

Experimental Design

All subjects performed a passive-viewing task. An infrared camera (EyeLink II, SR Research) monitored the position of the subject's gaze. Subjects began trials by fixating on a 0.7° fixation dot within a 3° window for between 200-300ms. The rendered face or object stimulus was then presented for 500ms with 500ms inter-stimulus interval in trials of 3 stimuli and all trials were aborted if the subject broke fixation for more than 100ms. The virtual avatar was positioned such that the cyclopean eye (the midpoint between the eyes) always remained at the center of the screen All stimuli were presented in side-by-side stereoscopic 3D on an OLED 3D TV (LG). A pair of 3D printed goggles holding polarized lenses was positioned in front of the subjects to create disparity and enable 3D presentation. All stimuli were presented using a graphical user interface (GUI) modified and derived from PLDAPS [3] in MATLAB.

When presenting the macaque avatar, we initially positioned the 3D TV screen at a viewing distance of 95cm from the subject. We initially presented 9 absolute sizes ranging from 8.7 cm, approximately two-thirds the size of an average macaque face, to 17.3 cm, approximately four-thirds the size of an average macaque face separated by equal increments. Each of these sizes was presented at 9 virtual distances, ranging from 63.3cm to 126.7cm for a total of 81 stimuli. Importantly, these sizes and distances were matched such that 9 stimuli would subtend the same retinal angle but differ in their physical properties, enabling us to disentangle physical size from retinal size (**Fig 1A**, **1B**). Extending this approach further in the second set of experiments, we created 20 absolute sizes of the avatar ranging from 1.3cm (one tenth the size of an average

macaque face) to 26 cm, (double the size at increments of 1.3. The OLED monitor was positioned at 90cm in front of the animal, with stereoscopic cues dictating the virtual distance of each stimulus as well as its size and volumetric shape.

Aside from the macaque avatar, we also evaluated tuning of AF neurons for several additional 3D rendered animals and objects. These included familiar objects (banana, apple), unfamiliar objects (fork, cluster of rocks, soda bottle, house, watermelon) and unfamiliar animals (elephant, goat, butterfly) (**Fig S3**). These objects were selected to provide a range of shapes and real-world sizes. Each object and animal, including the. macaque avatar was stereoscopically displayed at nine physical sizes: 1.3cm, 5.2cm, 7.8cm, 10.4cm, 13cm, 15.6cm, 18.2cm, 20.8cm, and 26cm. For this experiment, stimuli were rendered volumetrically and presented at a single virtual distance of 90cm.

In addition to main experimental stimuli, we presented "fingerprinting" stimuli at the beginning of each session, containing 60 images with face (human faces and monkey faces) and non-face (scenes and objects) categories.

Electrophysiological Recordings

Following fMRI localization, the AF face patch in both monkeys received an implant of a 64 channel NiCr microwire bundle fabricate by Microprobes for extracellular recording. Following implantation, the microwire was advanced towards AF face patch and its positioning confirmed with further MRI scans (**Fig 1C**). All recordings were conducted in a radio shielded room (ETS-Lingreen) with a RZ2 BioAmp processor (Tucker-Davis Technologies) with a 128-channel capacity collecting a broadband signal of 0.5Hz-20KHz.

Data Analysis

All data was analyzed in custom software designed in MATLAB. All spike sorting was conducted offline. All spike data was automatically sorted using the wave_clus [4] spike sorting package with the resources of the NIH HPC Biowulf cluster (http://hpc.nih.gov). We calculated the mean spike rate for each neuron for each stimulus in a window from 50ms to 500ms after stimulus onset and subtracted a baseline value calculated from the mean spike rate between 200ms and 50ms before the presentation of stimulus and used it for further analysis. For normalized plots including tuning curves and spike density functions, we divided all responses for each neuron by

the peak responses across all stimuli. For the initial set of nine sizes and distances, we used these responses to conduct a two-way ANOVA for each cell to determine the significance of the physical factors and to a conduct a one-way ANOVA to determine if the responses of neurons to the equal retinal angle stimuli changed significantly between stimuli of the same retinal subtence.

For this set, we also used these responses to calculate the model fit and model preference for each neuron to assess the relative contribution of size and retinal angle to the responses of neurons. To examine this, we first calculated the deviance of a linear multiple regression analysis of each cells including both physical size and retinal angle as factors. We then contrasted this value with the deviance of independent models for physical size and retinal angle with the following formula (equation 1):

$$\text{Model Fit} = 100\% * (D_{cons} - D_{test})/(D_{cons} - D_{Full}) ,$$

where D_{cons} is the deviance of a constant model of neural responses, D_{Full} is the deviance of the full multiple regression with physical size and retinal angle as factors, and D_{test} is the deviance of a single-factor regression with either physical size or retinal angle as a factor. From the model fit of both factors, we calculated the model preference (equation 2):

$$\text{Model Preference} = \text{Model Fit}_{phys} - \text{Model Fit}_{dva},$$

where Model Fit_{phys} is the fit relative to the physical size parameters and Model Fit_{dva} is the fit to the degrees visual angle parameters. A positive Model Preference indicates physical size explains most of the variance of the model whereas a negative Model Preference indicates retinal angle explains more of the variance.

Each neuron was separately evaluated for its face selectivity index. (FSI): (equation 3):

$$\text{FSI} = (R_{face} - R_{nonface})/(R_{face} + R_{nonface}),$$

where R_{face} is the average spiking rate response to face images and $R_{nonface}$ is the average spiking rate response to nonface images (for a plot of these look to **Fig 1D**) FSI was computed based on the response to a fixed set of 60 face and non-face “fingerprinting” stimuli, described above.

Supplemental Figure

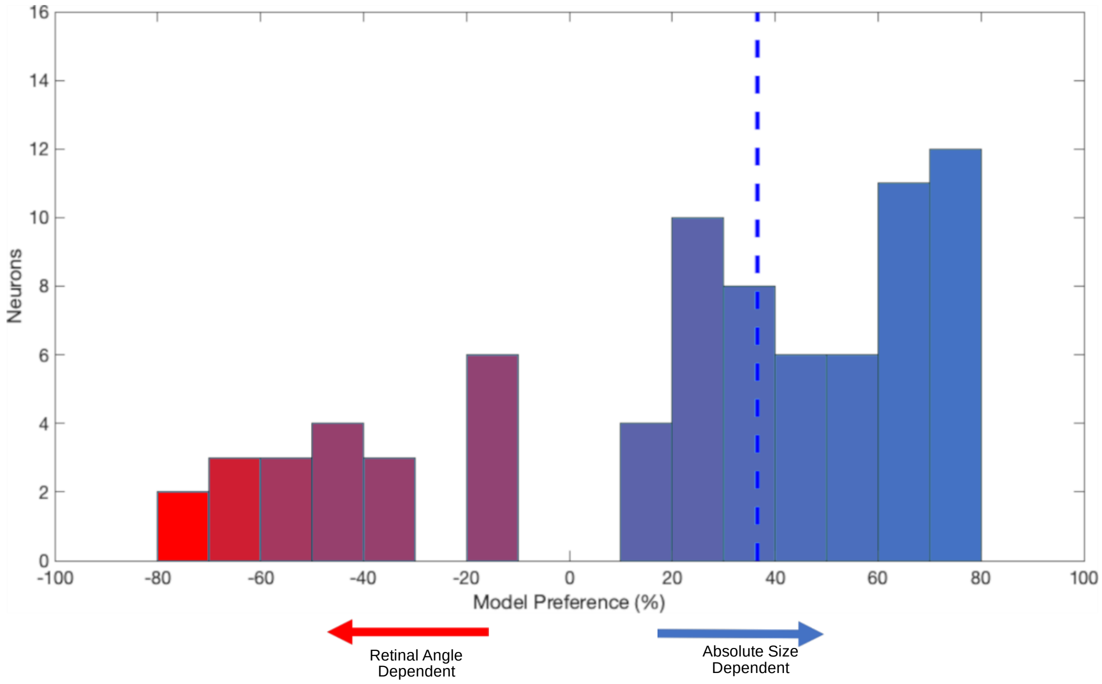

Figure S1 Related to Figure 3. Model preference of face selective neurons. A histogram of the model preference of the face selective neurons of the population (n=78). The dashed blue line displays the median of the population (36.6%) and indicates that even face selective neurons predominately respond to absolute size as opposed to retinal angle

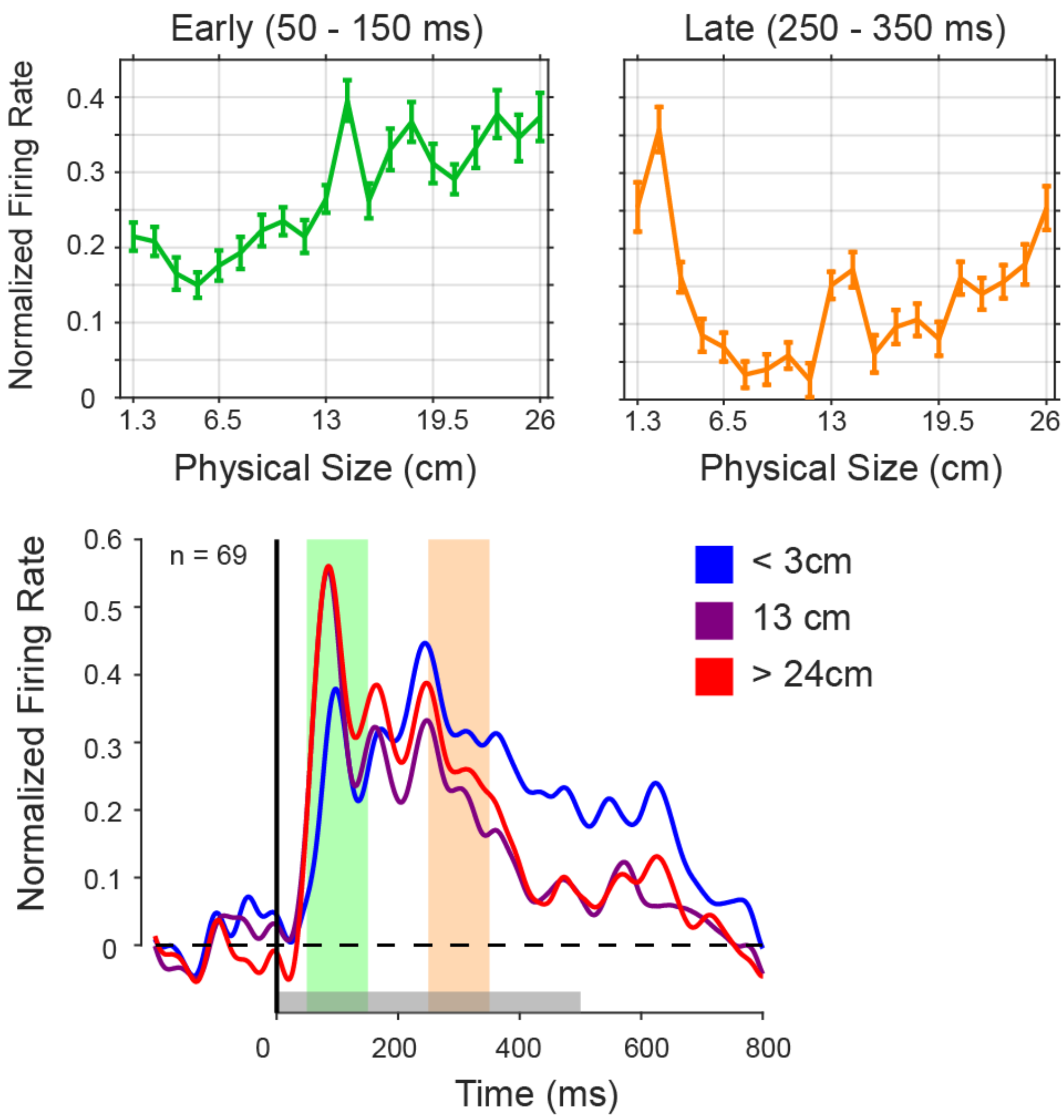

Figure S2 Related to Figure 4 and Figure 5.Time course for extreme size tuning. the average time course of neurons to the average of the largest 2 sizes in red, the smallest two in blue, and the middle size in purple with average tuning curves across all neurons for different sliding time windows for the time indicated by the color of the shaded windows

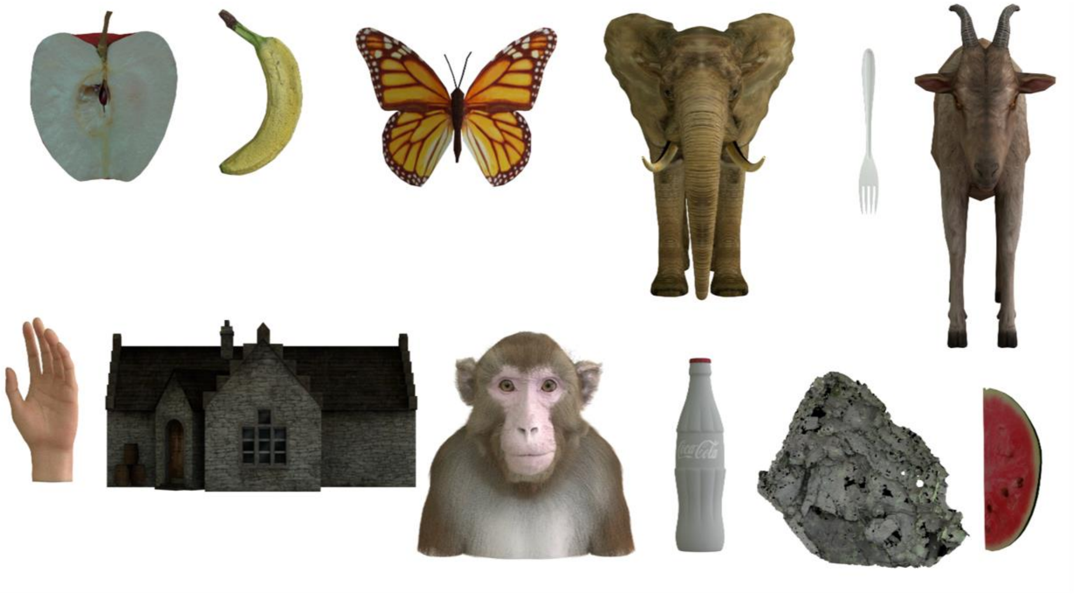

Figure S3 Related to Figure 5. 3D object stimuli. The object stimuli matched to the size of the macaque face (from crown to chin) and presented to subjects as part of the same image set. The animal stimuli with faces were also matched in size with the macaque face. These stimuli were all presented in 3D to examine if different images or objects would similarly show tuning for the larger sizes of the macaque face.

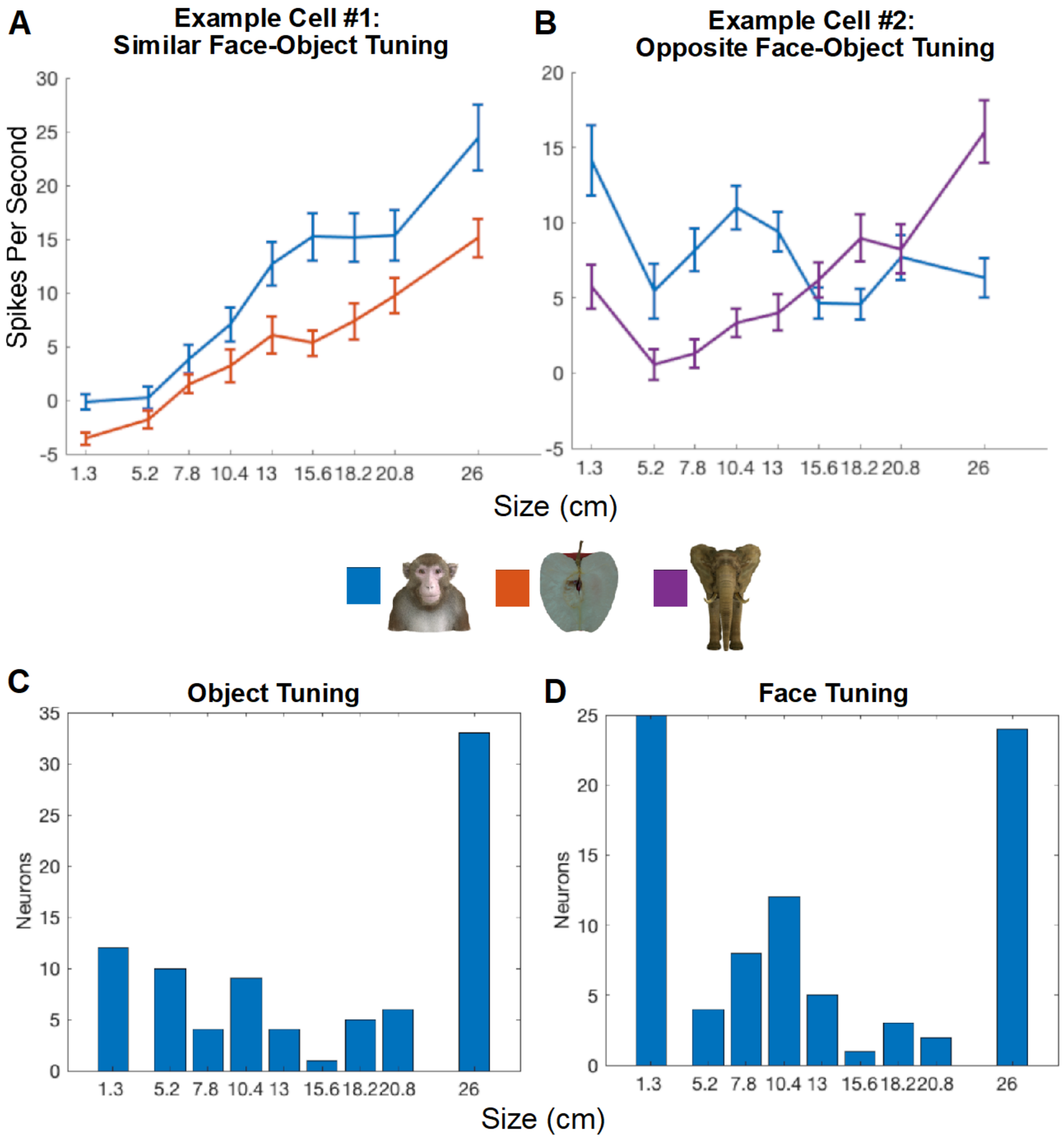

Figure S4 Related to Figure 5. Neural responses to 3D object stimuli. A. Tuning plot of a neuron, which shows similar extreme size tuning between face and the most stimulating object, an apple. B Heatmap of a neuron with extreme size tuning the most stimulating object (an elephant) uncorrelated with the size tuning of the face. C, D the size that yields the maximum response for same population of neurons neuron for the most stimulating object and the macaque face stimulus, respectively

References (Materials and Methods)

1. Koyano, K.W., et al., *Dynamic Suppression of Average Facial Structure Shapes Neural Tuning in Three Macaque Face Patches.* Curr Biol, 2021. **31**(1): p. 1-12 e5.
2. Russ, B.E. and D.A. Leopold, *Functional MRI mapping of dynamic visual features during natural viewing in the macaque.* Neuroimage, 2015. **109**: p. 84-94.
3. Eastman, K.M. and A.C. Huk, *PLDAPS: A Hardware Architecture and Software Toolbox for Neurophysiology Requiring Complex Visual Stimuli and Online Behavioral Control.* Front Neuroinform, 2012. **6**: p. 1.
4. Quiroga, R.Q., Z. Nadasdy, and Y. Ben-Shaul, *Unsupervised spike detection and sorting with wavelets and superparamagnetic clustering.* Neural Comput, 2004. **16**(8): p. 1661-87.
